# The nonlinear impact of ESG performance on audit pricing: Evidence from China

**DOI:** 10.1371/journal.pone.0331504

**Published:** 2025-09-04

**Authors:** Yajin Li, Fei Meng

**Affiliations:** 1 Accounting School, Capital University of Economics and Business, Beijing, China; 2 Shanghai Institute of Justice, Shanghai University of Political Science and Law, Shanghai, China; Ovidius University of Constanta: Universitatea Ovidius din Constanta, ROMANIA

## Abstract

The substantial impact of corporate Environmental, Social, and Governance (ESG) performance, predominantly measured by non-financial metrics, on external auditors is investigated in this paper. Using a sample of Chinese-listed companies from 2011 to 2021, we examine how auditors respond to corporate ESG performance from the perspective of audit pricing. The findings reveal a U-shaped, non-linear relationship between corporate ESG performance and audit pricing. Enhancing ESG performance significantly reduces audit pricing up to a certain threshold, but the impact diminishes as performance continues to improve. Internal control quality and the degree of information asymmetry play pivotal moderating roles within this U-shaped relationship; higher-quality internal controls and lower information asymmetry contribute to flattening the U-shaped curve. Mechanism analysis illustrates that ESG performance influences audit pricing by mitigating financial and operational risks. This paper supplements the understanding of ESG’s impact on audit pricing from a non-linear perspective, offering valuable insights for companies actively engaged in ESG practices.

## 1. Introduction

Sustainable development is currently a shared global pursuit, with an increasing number of companies embedding key elements of ESG (Environmental, Social, and Governance) into their governance, strategies, risk management, and goals. On June 26, 2023, the International Sustainability Standards Board (ISSB) formally released the definitive versions of two International Financial Reporting Sustainability Disclosure Standards: IFRS S1 (General Requirements for Sustainability-Related Financial Information Disclosure) and IFRS S2 (Climate-Related Disclosures); On July 31, 2023, the European Commission released 12 Sustainable Development Reporting Standards (ESRS). This marks a new phase in global sustainability information disclosure, signifying the entry of ESG disclosure into an advanced stage. ESG disclosure, primarily based on non-financial information, has a significant impact on external auditors. Traditionally, auditors have acted as ‘gatekeepers’ for targeted companies based on financial information. However, as leading enterprises adopt ESG principles, external auditors are increasingly focusing on a company’s ESG performance to comprehend its ESG practices and respond accordingly [1]. With the trend of integrating ESG and financial reports, the impact of corporate ESG performance on the audit domain has become an important theoretical and practical issue. In the risk-based audit model, corporate ESG performance significantly influences audit market pricing decisions [2].

The auditor’s work can be viewed as a risk compensation mechanism wherein they accept audit fees from the audited entity, enhancing the accuracy and authenticity of the company’s financial reports and offering assurance to their users [3]. Any deviation from strict compliance with guidelines or instances of negligence during the audit process, leading to the issuance of inaccurate audit opinions, can significantly impact the audit firm adversely. Consequently, within a risk-based audit framework, when the audited entity carries higher risks, the audit firm tends to employ more intricate audit procedures, incurring elevated operational costs, hence necessitating augmented audit fees.

Chinese companies’ ESG practices are in a phase of rapid development, and existing research has highlighted the economic consequences stemming from various ESG domains. Operationally, ESG initiatives have the potential to augment employee productivity, optimize investment structures, and curtail production expenses, thereby elevating a company’s financial performance [4]. In terms of corporate value, robust ESG performance can enhance both enterprise value and profitability [5]. Regarding financing costs, elevated social responsibility reporting correlates with reduced equity financing expenses. Companies assuming more social responsibility can leverage lower costs of equity capital [6]. Assessing risk levels, a corporation’s strong ESG performance significantly diminishes default risks while fostering a positive corporate reputation [7]. Improvements in a company’s financial standing through ESG performance can diminish the risk premium in certain audit pricing components [8], thereby influencing the audit fees charged by auditors.

The majority of existing research focuses more on the economic ramifications of corporate ESG performance, paying less attention to its impact on external audit behaviors. Research on audit pricing matters has tended to incorporate non-financial dominant ESG performance to a lesser extent. The current parallel research paths to some extent neglect the emerging trend of integrating non-financial and financial data disclosures. Among the few studies exploring the relationship between ESG performance and audit pricing, Song et al. [8] noted a negative correlation between ESG performance and audit pricing, mainly driven by S and G pillars. Xiao et al. [9] found that publicly disclosing ESG ratings could decrease audit pricing. Existing studies predominantly suggest a linear relationship, indicating a negative correlation between corporate ESG performance and audit pricing. This suggests that favorable ESG performance reduces audit pricing, while overlooking any potentially negative impacts or non-linear relationships. In the context of China’s emerging capital market, where the legal framework is still under development and lacks robustness [10], will audit fees also decline in a sustained manner as the level of corporate ESG continues to improve? In other words, does the positive impact of good ESG performance on lowering audit fees exhibit a diminishing marginal effect?

Based on this, the paper focuses on exploring the nonlinear relationship between corporate ESG performance and audit pricing. Using a sample of Chinese listed companies from 2011 to 2021, it analyzes the impact of ESG performance on audit pricing, investigating the pathways through internal control, information disclosure, and risk perspectives to understand ESG’s role in audit pricing. The marginal contributions of this paper include the following: (1) Supplementing research on factors affecting audit pricing, extending the study of ESG effects, and providing a partial basis for auditors to mitigate audit risks and enhance audit efficiency. (2) Exploring the boundaries of the effects of internal control and information disclosure on ESG’s reduction of audit pricing, guiding listed companies on improving ESG practices. (3) Establishing an intermediary mechanism model by selecting financial risk and operational risk to unravel how ESG performance influences audit pricing.

The rest of the article is organized as follows. In Section 2, we provide a literature review and discuss the research hypotheses. In Section 3, we introduce the research design. The main empirical results are presented in Section 4, and we present the conclusions and discussion in Section 6.

## 2. Literature review and hypothesis development

Existing research has extensively analyzed factors influencing audit pricing from perspectives such as individual companies, audit institutions, and external environments. At the individual company level, governance structures, internal controls, CEO characteristics, and religious beliefs all impact audit pricing [11–13]. Elevated corporate risks lead to increased demands in audit pricing [14], while performance-based managerial compensation schemes within companies have been linked to escalated audit costs [15]. In the United States, religious beliefs contribute to a more conservative management approach, with a tendency toward risk aversion. Auditors perceive the audit risks and audit work costs for such clients as lower, which is reflected in audit pricing [16]. Companies that do not adhere to social norms are charged higher audit fees [17]. Additionally, research in different regions needs to consider varying influencing factors. Hu et al. (2023), based on a quasi-natural experiment from China’s anti-corruption campaign, found that a reduction in corporate corruption leads to lower audit fees, with this effect being more pronounced in regions with weaker legal environments [18]. Xiao et al., based on a Chinese sample, studied the impact of the 2015 ESG rating announcement by the China Securities Regulatory Commission (CSRC) on audit fees. The results showed that ESG ratings significantly reduce audit fees [9]. This conclusion is of significant support for the development of China’s ESG framework. From the perspective of audit institutions, factors like belonging to the Big Four and auditor characteristics significantly impact audit pricing. Larger corporates usually have higher audit fees [19]. Additionally, auditors possessing greater specialized knowledge and experience tend to demand higher fee premiums [20]. External environmental factors, including negative media coverage, notably increase corporate audit fees [21]. Macro environments also influence audit environment risks, during financial crises or times of substantial market pressure, audit pricing significantly rises [22–23].

Auditors evaluate client risk levels to gauge workload, potential risks, and potential losses, which are reflected in audit pricing. According to signaling theory, ESG information reflects a company’s non-financial condition. When a company has a low ESG score, it may signal operational or management issues, adversely impacting the company’s financing costs [24]. This implies higher audit risks for auditors. Simultaneously, auditors may need to invest more effort during the audit process to mitigate failure risks, delving deeper into the audited entity’s financial status and integrity concerns. Companies with strong ESG performance not only boost social recognition but also cultivate a positive reputation to uphold their competitive advantages. It also signifies their adeptness in effective communication and collaboration with stakeholders, and better cooperation with management in audit tasks, thereby enhancing some aspects of the auditor’s efficiency.

However, in practical terms, as ESG performance improves, an increase in each unit’s ESG performance may not necessarily lead to a proportional decrease in audit fees. Consequently, relying solely on a linear assumption to study the relationship between corporate ESG performance and audit pricing may have certain limitations.

With growing societal expectations regarding corporate social and environmental responsibilities, an increasing number of listed companies are employing impression management tactics. These include practices like “accentuating positives while concealing negatives” and “superficial gestures without substantive action,” which embellish companies’ non-financial performance. China’s capital market lacks “green efficiency” [25], especially amidst the early stages of ESG development in China. Consequently, varying degrees of “greenwashing” exist, where companies selectively disclose critical information to foster a positive social image while concealing adverse information [26–27]. This situation not only fails to aid financial information users but also compromises the integrity of financial reports themselves [28]. Consequently, auditors must exercise caution when faced with exceptionally high corporate ESG performance. They need to consider the potential opportunistic use of ESG ratings, strategic disclosure of redundant information, and the resultant amplification of information noise. These factors contribute to heightened complexity and risks in the auditor’s responsibilities [29].

Moreover, improving ESG performance requires associated costs. Companies must account for environmental costs during product production and various subsidies provided to employees. Excessive ESG investments might lead to diminishing returns, potentially lowering the company’s profitability and placing pressure on management to achieve targets. This could incentivize earnings manipulation and false financial reporting, thereby amplifying audit risks. An excessive focus on improving corporate ESG performance might divert attention from other aspects, potentially loosening resources and exacerbating agency problems, elevating operational risks for the company. Consequently, auditors might enhance their evaluation of audit risks, leading to higher audit fees. Therefore, the hypothesis is proposed:

**H1** There exists a U-shaped relationship between corporate ESG performance and audit pricing.

According to the Guidelines on Internal Control for Listed Companies issued by the China Securities Regulatory Commission (CSRC), internal control aims to constrain managerial self-interest through established systems, enhancing operational efficiency within organizations. During audit procedures, auditors focus on assessing control risks. If a company faces higher internal control risks, auditors must intensify their audit efforts. This involves altering audit procedures, engaging in extended discussions with company management, and incurring higher costs for substantive testing and significant decision-making time [30]. Companies with significant internal control deficiencies are inherently exposed to higher risks, posing potential threats to the auditor’s reputation [31]. Consequently, audit firms might demand higher risk premiums from such enterprises [32–33].

As a supervisory mechanism, higher-quality internal controls mitigate the risk of financial misstatements and bolster the credibility of audit evidence. Thus, robust internal controls can alleviate the potential negative impacts of ESG performance, reducing agency problems and enabling ESG performance to leverage its positive reputation effects more effectively. This mitigation helps alleviate the diminishing marginal effect on audit pricing.

Inadequate internal control within companies leads to lower accruals quality, affecting financial restatements and sustainability of earnings [34]. When companies encounter heightened internal control risks, auditors need to allocate more resources toward audit costs for evidence gathering, potentially heightening the sensitivity of the relationship between corporate ESG performance and audit pricing. High-quality ESG information can help auditors conserve resources, consequently reducing audit pricing. However, lower-quality internal controls also limit the boundary within which ESG performance operates. Despite the impact of factors like client industry and regulatory environments on audit pricing, the diminishing effect of corporate ESG performance on audit pricing persists. Therefore, the following hypothesis is proposed:

**H2** Internal control moderates the relationship between corporate ESG performance and audit pricing.

**H2a** Higher-quality internal control smoothens the U-shaped curve between corporate ESG performance and audit pricing.

**H2b** Higher-quality internal control shifts the turning point of the U-shaped curve between corporate ESG performance and audit pricing to the right.

According to the economic theory of asymmetric information, well-informed parties in market transactions generally grasp a company’s operational and financial status better than uninformed traders, who may suffer losses due to information disparities. The degree of asymmetric information significantly influences asset liquidity. Effective information disclosure by listed companies increases the efficiency of capital market information, aiding stakeholders in making critical decisions and deepening auditors’ comprehension of audited entities, thereby providing auditors with more comprehensive and accurate audit information [35]. Consequently, a company’s information environment closely intertwines with potential risks encountered by audit institutions [36].

From the perspective of information disclosure, companies with strong ESG performance typically exhibit superior disclosure environments [37]. ESG information, encompassing non-financial company performance, offers a fresh perspective for the public to observe a company’s operational status, reducing information asymmetry and enhancing the probability of detecting adverse news. With the prevalence of sustainability principles, non-financial reports such as ESG reports play an increasingly pivotal role in the capital market. Strong ESG information disclosure not only enriches the financial information contained in financial reports but also enhances users’ trust in the authenticity of financial data. Therefore, a favorable information environment assists auditors in assessing audit risks, reducing costs associated with information acquisition and processing, and avoiding potential auditing complexities, thereby enhancing audit efficiency and accuracy while reducing audit pricing and delays [38]. For instance, in communication with corporate management, companies with robust information disclosure receive greater management cooperation. In a favorable information disclosure environment, the mitigating effect of ESG performance on audit pricing can be strengthened.

Conversely, as the asymmetry of company information worsens, companies might be more inclined to conceal potentially adverse news or manipulate stock prices. In such cases, inaccurate disclosure of pivotal information creates substantial uncertainty for auditors, possibly intensifying their demand for ESG information and heightening the “information identification” effect of ESG. However, in unfavorable corporate information environments, the diminishing marginal effect of ESG on reducing audit pricing could be exacerbated by increased information asymmetry, manifesting more distinctly. In summary, the following hypothesis is proposed:

**H3** The degree of asymmetric information moderates the relationship between corporate ESG performance and audit pricing.

**H3a** Lower levels of asymmetric information tend to smoothen the U-shaped curve between corporate ESG performance and audit pricing.

**H3b** Higher levels of asymmetric information tend to shift the turning point of the U-shaped curve between corporate ESG performance and audit pricing to the left.

In the course of their work, auditors pay particular attention to various risks associated with the audited entity’s ongoing operations [39]. Signaling theory suggests that poor ESG performance emits signals of unhealthy business practices, raising investors’ concerns about uncertain prospects, escalating financing challenges, and heightening default and financial risks [40]. Conversely, companies with better ESG performance exhibit stronger resilience against risks [41]. They can navigate adverse environments more effectively, enhance financial stability, and facilitate the accumulation of critical resources. Improved ESG performance helps establish more stable relationships between companies and stakeholders, alleviating agency issues with major shareholders [42]. This aids in securing funding, reducing financial risks, and potentially reducing the auditor workload, thus lowering audit fees.

However, companies frequently embroiled in ESG-related controversies may face increased legal litigation risks, which could signal poor business conditions. Elevated operational risks increase the likelihood of encountering crises, potentially harming an auditor’s reputation and increasing the audit firm’s exposure to joint liabilities, thus raising audit risks. Confronted with potential company risks and issues, auditors may opt to intensify audit procedures, conduct more extensive testing, and gather additional evidence to enhance the likelihood of detecting significant misstatements or omissions, thus reducing the risk of audit failure [43]. According to the audit pricing model [2], higher audit costs correspond to higher audit fees. In summary, the following hypotheses are proposed:

**H4a** Improved ESG performance can reduce a company’s financial risks, consequently lowering audit pricing.

**H4b** Improved ESG performance can reduce a company’s operational risks, consequently lowering audit pricing.

## 3. Research design

### 3.1. Data sources and sample selection

This paper selects A-share corporate listed on the Shanghai and Shenzhen stock exchanges in China from 2011 to 2021 as the research sample to investigate the impact of corporate ESG performance on audit pricing. To avoid interference, the study excludes samples from the financial industry, as this sector typically exhibits different risk profiles and financial characteristics compared to other industries. Additionally, companies categorized under ST (Special Treatment: A label for listed companies with abnormal financial conditions in China’s A-share market),*ST (Special Treatment), SST (Special Special Treatment), and PT (Particular Treatment) statuses are excluded due to their potential financial instability, which could distort the analysis of audit pricing. Winsorization is applied to continuous variables to eliminate the influence of outliers. Data is sourced from the CSMAR and Wind databases, while internal control data originates from the internal control index published by Debo Enterprise Risk Management Company. We used Stata 17.0, provided by StataCorp LLC, for statistical analysis in this study.

### 3.2. Definition of variables

#### 3.2.1. Independent variable.

The independent variable is the corporate ESG performance, measured by Huazheng Development’s ESG rating index for listed companies. Huazheng Development is one of the early third-party institutions in China involved in ESG evaluations. Compared to other rating agencies, Huazheng’s coverage is broader, incorporating 26 key indicators within its evaluation system. The assessment system references mainstream international ESG frameworks, aligning with the practicalities of the Chinese market. It employs an industry-weighted average approach. Following previous research [44], the rating results “AAA-C” are scored from high to low as “9-1” to construct the explanatory variable.

#### 3.2.2. Dependent variable.

The dependent variable is audit pricing: following prior research, this paper adopts the natural logarithm of audit fees to measure audit pricing [45].

#### 3.2.3. Moderating variables.

This paper employs the DIB internal control index from the Debo database to gauge corporate internal control levels. Debo Company constructs nine sub-databases based on the five key elements of enterprise internal control, which include control environment, risk assessment, control activities, information and communication, and monitoring. These elements, as outlined in the COSO framework, help assess the effectiveness of a company’s internal control systems.

In measuring information asymmetry, this paper utilizes stock trading data obtained from listed companies in the Chinese capital market, precisely selecting daily frequency trading data for analysis. Specifically, the liquidity ratio (LR), illiquidity ratio (ILL), and the return reversal indicator (GAM) are subjected to principal component analysis [46–47]. Consistent with previous studies, the first principal component is extracted from the original indicators, removing components unrelated to information asymmetry [48]. This is denoted as the Asymmetry Index (ASY). A higher value indicates lower stock liquidity and more severe information asymmetry. The relevant equations are shown in Equations (1) and (2).


$$LR_{it}=-\frac{1}{D_{it}}\sum_{k=1}^{D_{it}}\sqrt{\frac{V_{it}(k)}{\left|r_{it}(k)\right|}}$$ (1)

$$ILL_{it}=\frac{1}{D_{it}}\sum_{k=1}^{D_{it}}\sqrt{\frac{\left|r_{it}(k)\right|}{V_{it}(k)}}$$ (2)

Among these, r_it_(k) represents the stock return of the company on the k trading day in i year t, V_it_(k) denotes the daily trading volume, and D_it_ indicates the number of trading days in the year. When liquidity is low, the value of ILL becomes larger, and due to the presence of the negative sign, the value of LR also increases. The return reversal indicator GAM=|rit|. The coefficient r_it_ is estimated using Equation (3).


$${r^{e}}_{it}(k)=\theta_{it}+\varphi_{it}r_{it}(k-1)+\gamma_{it}V_{it}(k-1)sign[{r^{e}}_{it}(k-1)]+\varepsilon_{it}(k)$$ (3)

Where ${r^{e}}_{it}(k)=r_{it}(k)-r_{mt}(k)$ is the excess return, $r_{mt}(k)$ stands for the market return weighted by market value. A larger GAM implies lower liquidity. $\theta_{it}$ represents the intercept term. $\varphi_{it}r_{it}(k-1)$ denotes the autocorrelation term of returns, where $\varphi_{it}$ is the autocorrelation coefficient reflecting the strength of the relationship between the current return and the previous period’s return. $\gamma_{it}V_{it}(k-1)sign[{r^{e}}_{it}(k-1)]$ Represents the liquidity reversal term, where $\gamma_{it}$ is the coefficient that measures the impact of trading volume on return reversal, and $V_{it}(k-1)$ indicates the trading volume of the previous period. The “sign” function in this equation is used to represent the direction of the excess return in the previous period and acts as a sign function. $\varepsilon_{it}(k)$ Is the random error term, which is used to capture the unexplained random variations in the model.

#### 3.2.4. Mediating variables.

This paper selects financing constraints as a proxy variable for financial risk. The greater the financing constraints, the higher the representation of financial risk for the company. Presently, methods for assessing corporate financing constraints mainly include the KZ index, WW index, and SA index. However, the underdevelopment of the Chinese capital market causes valuation biases in enterprises, impacting the applicability of the KZ index [49], and the WW index calculation involving endogenous variables such as cash flows, which might pose endogeneity issues. Hence, following Hadlock and Pierce’s research [50], the article employs the SA index to measure the degree of financing constraints. The computation method is presented in Equation (4).


$$SA=-0.737Size+0.043Size^{2}-0.040Age$$ (4)

Where Size represents the natural logarithm of the company’s size, and Age denotes the company’s establishment time, both having strong exogeneity, ensuring relatively more scientific results. A higher SA index indicates more significant financing constraints, signifying more severe financial risks for the company.

Past studies consistently indicate that higher operational risks in companies lead to higher profit volatility [51–52]. Therefore, this paper uses the degree of profit volatility to measure company operational risks. The relevant equations are illustrated in Equations (5) and (6).


$$\delta_{i,t}=\sqrt{\frac{1}{T-1}\sum_{t=1}^{T}{\left(E_{i,t}-\frac{1}{T}\sum_{t=1}^{T}E_{i,t}\right)}^{2}}|T=4$$ (5)

$$E_{i,t}=\frac{EBITDA_{i,t}}{A_{i,t-1}}$$ (6)

Where $\delta_{i,t}$ represents the operational risk of a company i in year t, $EBITDA_{i,t}$ stands for the earnings before interest, taxes, depreciation, and amortization (EBITDA) of company i in year t, and $A_{i,t-1}$ represents the total assets of company i in year t-1. This paper selects the rolling standard deviation of EBITDA margin from year t-4 to year t-1. Additionally, due to the non-normal distribution of results, this paper further computes the cumulative distribution probability of the EBITDA margin standard deviation to measure company operational risks.

#### 3.2.5. Controls variables.

Drawing from previous research [53], this article selects the following control variables: Company Size (Size), Leverage (Lev), Return on Assets (ROA), Auditor Size (Big4), Auditor Tenure (AudTen), Auditor Change (Ach), Prior Audit Opinion Type (LagOP). The specific variable definitions are presented in Table 1.

**Table 1:** Variable definitions and summary descriptions.

	Variable Name	Variable Symbol	Variable Measurement
Independent Variable	ESG Performance	ESG	ESG Scored from “9-1” based on Huazheng ESG Evaluation System
Dependent Variable	Audit Pricing	LnFEE	Natural logarithm of audit fees
Moderating Variables	Internal Control Quality	IC	Di Bo’s published Internal Control Index, higher values indicate better internal control quality, normalized by dividing by 100
	Information Asymmetry	ASY	Computed using Principal Component Analysis, larger values indicate higher levels of asymmetry
Mediating Variables	Financial Risk	SA	Financial constraints
	Operational Risk	Risk	Profit volatility
Control Variables	Company Size	Size	Natural logarithm of total assets at year-end
	Leverage Ratio	Lev	Total liabilities at year-end divided by total assets at year-end
	Return on Assets	Roa	Net income divided by average total assets
	Auditor size	Big4	which is equal to one if the auditor is international Big 4 and zero otherwise
	Auditor Change	Ach	Assigned a value of one for auditor changes, otherwise zero
	Auditor Tenure	AudTen	Audit tenure in years for the auditor firm
	Previous Audit Opinion Type	LagOP	Value of one for standard audit opinion, otherwise zero

These control variables reflect various factors that may influence audit pricing. For example, company size (Size) is often considered one of the key determinants of audit fees, as larger companies typically require more audit resources. Similarly, leverage (Lev) and return on assets (ROA) are critical indicators for measuring a company’s financial risk and profitability, which may affect audit fees. In addition, factors such as the size of the audit firm (Big4) and audit firm change (Ach) may also impact audit fee pricing, as larger firms typically involve higher audit costs, and a change in audit firm may signify new audit risks. Additionally, the article controls for the year (Year) and firm (Firm) influences to account for heteroscedasticity in the time-series data and potential cross-sectional biases.

### 3.3. Model construction

This paper concurrently controls for individual and yearly fixed effects. To alleviate the issue of endogeneity and bidirectional causality in the model, it includes the lagged explanatory variable. Additionally, the model introduces the squared term of ESG performance (ESG^2^) to examine the nonlinear relationship between corporate ESG performance and audit pricing. A baseline regression model was established as shown in Equation (7).


$$LnFEE=\alpha_{0}+\alpha_{1}ESG+\alpha_{2}ESG^{2}+\alpha_{3}Controls+\sum Year+\varepsilon$$ (7)

Furthermore, the article explores the moderating effects of internal control and information asymmetry on the relationship between corporate ESG performance and audit pricing. We constructed Equations (8) and (9) to conduct the examination.


$$\begin{array}{c} LnFEE=\alpha_{0}+\alpha_{1}ESG+\alpha_{2}ESG^{2}+\alpha_{3}Controls+\alpha_{4}IC+\alpha_{5}ESG*IC\\ \;\;\;\;\;\;\;\;\;\;\;\;\;\;\;+\alpha_{6}ESG^{2}*IC+\sum Year+\varepsilon \end{array}$$ (8)

$$\begin{array}{c} LnFEE=\alpha_{0}+\alpha_{1}ESG+\alpha_{2}ESG^{2}+\alpha_{3}Controls+\alpha_{4}ASY+\alpha_{5}ESG*ASY\\ \;\;\;\;\;\;\;\;\;\;\;\;\;\;\;+\alpha_{6}ESG^{2}*ASY+\sum Year+\varepsilon \end{array}$$ (9)

Within the mechanism analysis, the model is set as shown in Equations (10) and (11), and the regression coefficients are tested accordingly.


$$M=\alpha_{0}+\alpha_{1}X+\alpha_{2}X^{2}+\varepsilon_{i,t}$$ (10)

$$Y=\beta_{0}+\beta_{1}X+\beta_{2}X^{2}+\beta_{3}M+\varepsilon_{i,t}$$ (11)

Where M represents the mediating variable, X represents corporate ESG performance, and Y represents audit pricing.

## 4. Empirical analysis

### 4.1. Descriptive statistics

Table 2 reports descriptive statistics of the main variables. The ESG standard deviation is 1.112, indicating significant variation in ESG performance across companies. The mean audit pricing is 13.816, with a median of 13.710, suggesting a relatively symmetric distribution. All other control variables fall within reasonable ranges.

**Table 2:** Descriptive statistics of main variables.

Variable	N	Mean	Median	Std.	Min	Max
ESG	17327	4.1901	4.000	1.112	1.000	8.000
LnFEE	17122	13.816	13.710	0.759	12.542	16.965
IC	16957	6.434	6.651	1.214	0.000	9.954
ASY	17327	−0.069	−0.014	0.322	−1.341	0.841
SA	16724	−3.798	−3.807	0.269	−5.646	−2.109
Risk	12683	0.468	0.459	0.283	0.000	1.000
Size	17327	22.247	22.012	1.3656	19.525	26.430
Lev	17327	0.422	0.414	0.208	0.031	0.925
ROA	17327	0.041	0.040	0.066	−0.398	0.254
Big4	17327	0.072	0.000	0.259	0.000	1.000
Ach	17327	0.102	0.000	0.303	0.000	1.000
AudTen	17319	7.543	6.000	5.612	1.000	33.000
LagOP	14542	0.979	1.000	0.143	0.000	1.000

Before testing the benchmark model, we conducted a multicollinearity test on the core variables. All the VIF values in the test results are below 10, which indicates that there is no problem of multicollinearity among the core variables.

### 4.2. Regression analysis

Prior to conducting the baseline regression, the study conducted multicollinearity diagnostics on core variables to mitigate coefficient estimation errors caused by high inter-variable correlations. As reported in Table 3, The average VIF is 1.13, approximating the ideal value of 1. Following Vatcheva et al. [54], we adopted the dynamic threshold method where: max VIF = 1/(1-R^2^) = 2.19. (Derived from the model’s R^2^). All variables’ VIF values fall below 2.19. Therefore, the model exhibits no evidence of multicollinearity issues.

**Table 3:** Multiple Collinearity Test Results.

	VIF
ESG	1.14
ESG^2^	1.11
Size	1.14
Lev	1.25
ROA	1.13
Big4	1.00
Ach	1.18
AudTen	1.18
LagOP	1.05
Mean VIF	1.13

In Table 4, Column (1) reports the regression results based on the baseline model (7). It can be observed that controlling for relevant variables, the regression coefficient of the first-order term of corporate ESG performance is −0.080, significantly positive at the 1% level. The regression coefficient of the second-order term is 0.007, also significantly positive at the 1% level. These results suggest a nonlinear correlation, specifically a U-shaped relationship, between corporate ESG performance and audit pricing.

**Table 4:** Regression Analysis Results.

	(1)	(2)	(3)
**LnFEE**	**LnFEE**	**LnFEE**
ESG	−0.080*** (0.014)	−0.047^***^ (0.015)	−0.085^***^ (0.014)
ESG^2^	0.007*** (0.002)	0.004^*^ (0.002)	0.008^***^ (0.002)
IC		−0.011^**^ (0.005)	
ESG*IC		0.034^**^ (0.015)	
ESG^2^*IC		−0.003^*^ (0.002)	
ASY			−0.061^***^ (0.018)
ESG*ASY			−0.098^***^ (0.048)
ESG^2^*ASY			0.014^***^ (0.005)
Size	0.345***	0.305^***^	0.337^***^
	(0.009)	(0.007)	(0.009)
Lev	−0.024	−0.010	−0.018
	(0.038)	(0.036)	(0.039)
ROA	−0.795***	−0.690^***^	−0.823^***^
	(0.053)	(0.050)	(0.053)
Big4	0.356***	0.287^***^	0.355^***^
	(0.037)	(0.030)	(0.037)
Ach	−0.004	−0.003	−0.003
	(0.010)	(0.008)	(0.010)
AudTen	0.003***	0.004^***^	0.003^***^
	(0.001)	(0.001)	(0.001)
LagOP	−0.114***	−0.085^***^	−0.112^***^
	(0.023)	(0.022)	(0.023)
Year FE	Yes	Yes	Yes
Firm FE	Yes	Yes	Yes
cons	6.171***	7.003^***^	6.360^***^
	(0.184)	(0.148)	(0.195)
N	14442	14440	14442
R^2^	0.675	0.654	0.676

^a^Notes: The values within parentheses are standard errors clustered by company; ***, **, and * represent statistically significant at the level of 1%, 5%, and 10%.

As per the studies by Lind and Mehlum [55] and Haans et al. [56], a significant coefficient for the quadratic term alone isn’t enough evidence to confirm the existence of a U-shaped relationship. Further testing is necessary. According to the U-test results in Table 5, the signs of the slopes on either side of the turning point (5.558) differ. In the left interval (1, 5.558), the slope is −0.065, significant at the 1% level. Conversely, in the right interval (5.558, 8), the slope is 0.035, also significant at the 1% level. Overall significance at the 1% level confirms the existence of a U-shaped relationship.

**Table 5:** Presents the U-test results for assessing the U-shaped relationship.

	Left Interval (1, 5.558)	Right Interval (5.558, 8)
Slope	−0.065	0.035
t-value	−6.197	2.343
P > | t |	0.0000	0.010

^b^Notes: The upper and lower bounds in the table represent the interval boundaries of the U-test results. Specifically, Boundary 1 indicates the lower limit of the data within this range, while Boundary 8 represents the upper limit of the data range.

**Fig 1:**
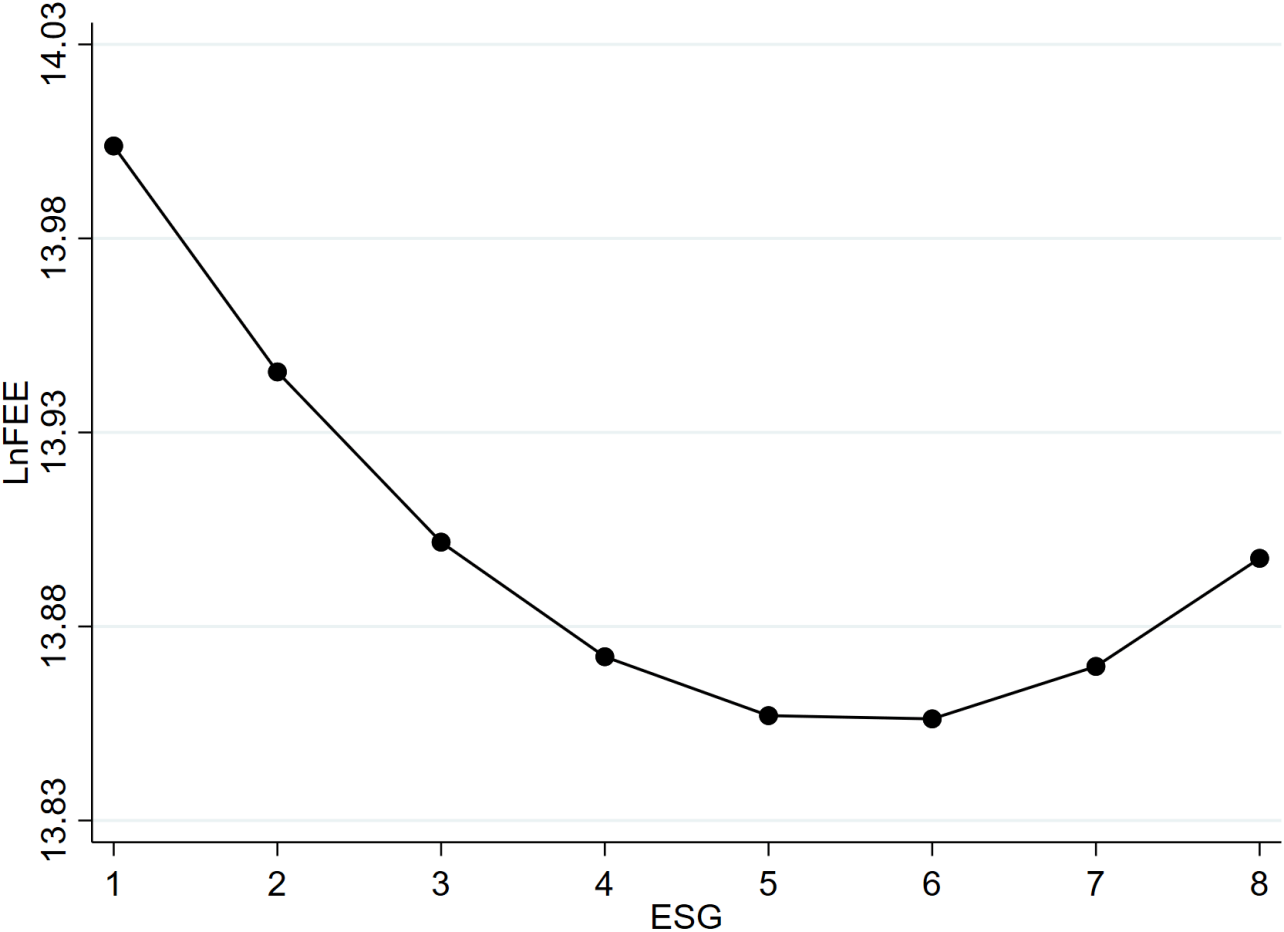
The U-shaped relationship between corporate ESG and audit pricing.

The graphical representation of this relationship is depicted in Fig 1. When ESG performance is below the turning point, an improvement significantly reduces audit fees. However, beyond 5.558, there is a reversal in their relationship, showing a diminishing effect of ESG on reducing audit fees. There exists an optimal level of ESG performance that minimizes the audit pricing set by certified public accountants. Therefore, Hypothesis 1 is confirmed, indicating that there is a U-shaped relationship between corporate ESG performance and audit pricing.

### 4.3. Analysis of moderating effects

#### 4.3.1. Moderating effect of internal control.

This paper further introduces internal control as a moderating variable, incorporating interaction terms between the moderating variable and the explanatory variable, as well as interaction terms between the moderating variable and the squared explanatory variable. This aims to investigate the moderating role of internal control in the U-shaped relationship between ESG performance and audit pricing. Before generating interaction terms, both the explanatory variable ESG and the moderating variable internal control quality (IC) undergo centralization to mitigate multicollinearity issues. The results in Column (2) of Table 4 indicate significance: the interaction between ESG and IC is positive at the 5% level, measuring 0.034, while the coefficient for IC and ESG^2^ interaction is negative at the 10% level, standing at −0.003. This suggests a significant moderating effect of internal control in the relationship between corporate ESG performance and audit pricing.

**Fig 2:**
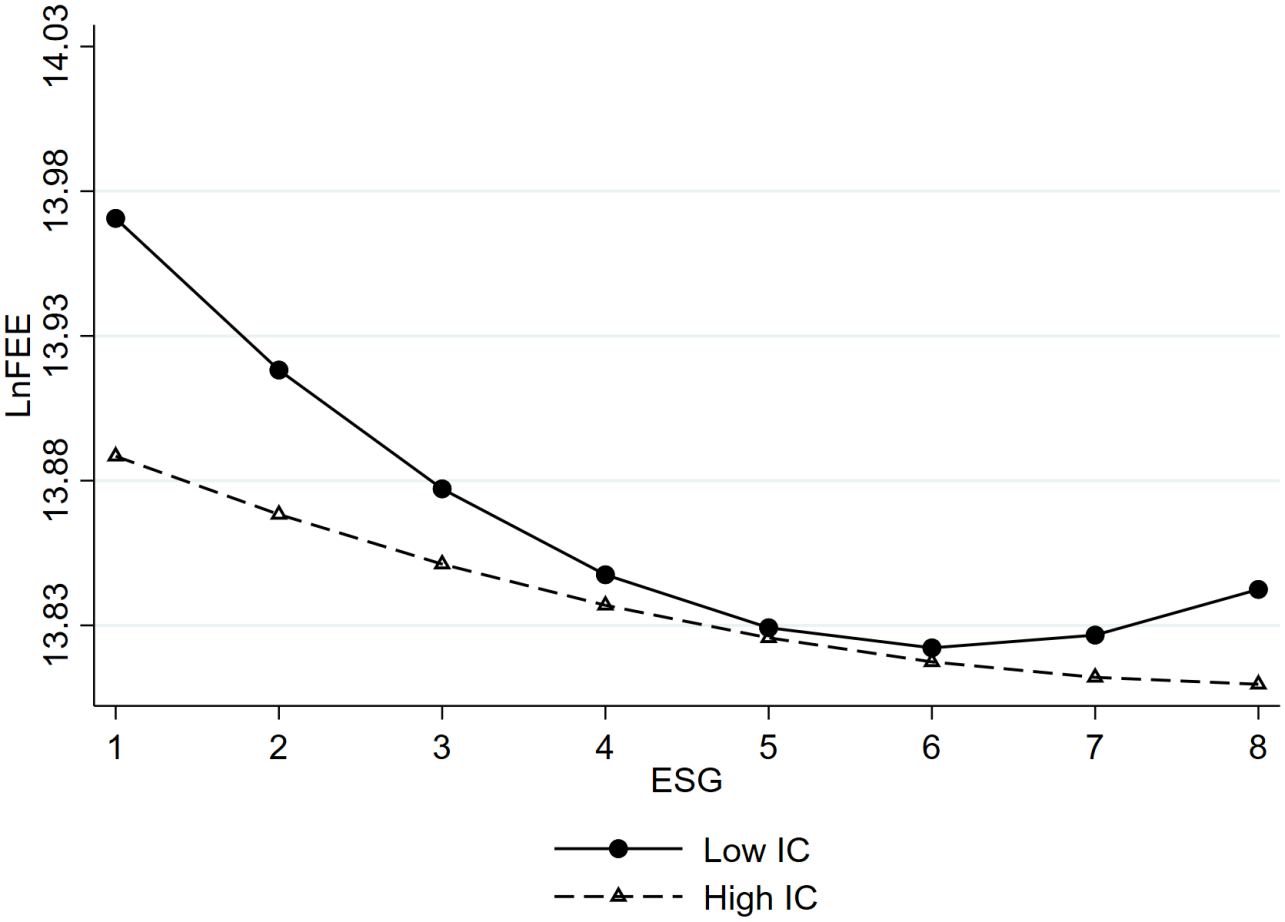
The moderating effect of internal control quality. ^c^Notes: To visually show the moderating effects, we divided the moderator variables into two groups. Low IC: IC = mean – standard deviation (6.541 - 0.660 = 5.881). High IC: IC = mean + standard deviation (6.541 + 0.660 = 7.201).Additionally, higher-quality internal controls shift the turning point of the curve to the right, implying that companies with lower internal control quality require a more substantial improvement in ESG performance to reach an optimal point.

The impact effects of this moderation can be observed in Fig 2. It’s evident that when internal control quality is low, the U-shaped curve between corporate ESG performance and audit pricing steepens. Conversely, in companies with higher internal control quality, the U-shaped curve is less pronounced. This indicates that within companies with inherently robust internal controls, the impact of ESG is relatively weak, suggesting that internal control quality weakens the relationship between ESG performance and audit pricing. Therefore, Hypothesis H2a is confirmed. The relative groupings of “Low IC” and “High IC” are based on a comparative approach rather than specific statistical thresholds. Here, “Low IC” refers to firms with relatively weaker internal control quality, while “High IC” refers to firms with stronger internal control quality. (Same for ASY below.)

#### 4.3.2. Moderating effect of information asymmetry.

As per Model (9), incorporating information asymmetry as a moderating variable, both the moderating variable ASY and the explanatory variable ESG are centralized before generating interaction terms to avoid multicollinearity issues. The outcomes in Column (3) of Table 4 reveal significance: the interaction between ESG and ASY is negative at the 5% level, measuring −0.061, while the coefficient for ASY and ESG^2^ interaction is positive at the 5% level, standing at 0.014. This indicates that information asymmetry significantly strengthens the relationship between corporate ESG performance and audit pricing.

**Fig 3:**
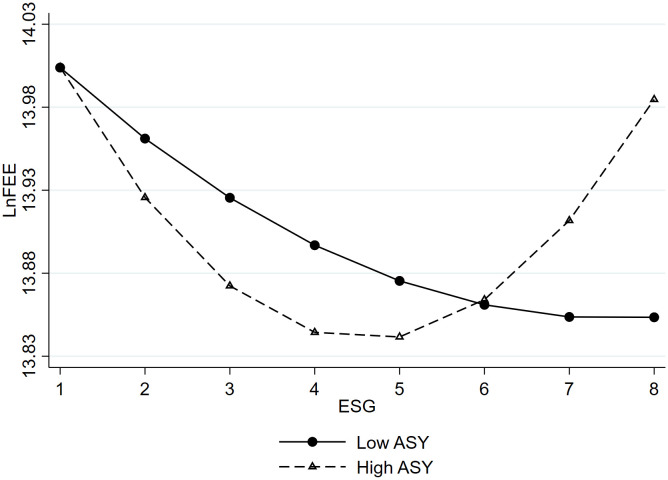
The moderating effect of information asymmetry. ^d^Notes: To visually show the moderating effects, we divided the moderator variables into two groups. Low ASY: ASY = mean – standard deviation (−0.069–0.322 = −0.391). High ASY: ASY = mean + standard deviation (−0.069 + 0.322 = 0.253).

Based on this, we depict a moderation effect graph as shown in Fig 3. As the degree of information asymmetry increases, the curve relating ESG to audit pricing becomes steeper. Therefore, the degree of information asymmetry moderates the relationship between ESG performance and audit pricing. Hypothesis H3a is confirmed, suggesting that lower information asymmetry leads to a flatter U-shaped curve between ESG performance and audit pricing. Under the moderating effect of information asymmetry, the turning point of the U-shaped curve shifts leftward to 5.251. indicating that when a company experiences higher information asymmetry, the optimal point of ESG performance’s effect on audit pricing shifts to the left. This confirms Hypothesis H3b. Therefore, if companies aim to reduce audit pricing through better ESG performance, they should work to mitigate the degree of information asymmetry between the company and the audit firm, thereby enhancing the effect of ESG performance in lowering audit pricing.

### 4.4. Analysis of mediating effects

#### 4.4.1. Examination of financial risk mediation effect.

In line with Hypothesis H4a, regression is conducted in Models (10) and (11) using financial risk (SA) as a mediating variable to explore whether financial risk mediates the relationship between ESG performance and audit pricing. Results in Column (1) of Table 6 reveal a significantly positive regression coefficient for the quadratic term of corporate ESG performance at the 1% level. Simultaneously, the coefficient for the variable ESG is significantly negative at the 1% level, indicating a U-shaped relationship between corporate ESG performance and financial risk. In Column (2), the mediating variable SA is introduced, and the results show that the coefficient of the variable ESG^2^ remains significant. Additionally, the mediating variable SA demonstrates a positive significant correlation with audit pricing LnFEE at the 1% level, implying that corporate financial risk partially mediates the U-shaped relationship between corporate ESG performance and audit pricing. Hypothesis H4a is confirmed.

**Table 6:** The examination of the intermediary effect.

	(1)	(2)	(3)	(4)
	SA	LnFEE	Risk	LnFEE
ESG	−0.009^***^	−0.075^***^	−0.078^***^	−0.064^***^
	(0.003)	(0.014)	(0.013)	(0.016)
ESG^2^	0.002^***^	0.006^***^	0.007^***^	0.005^**^
	(0.000)	(0.002)	(0.002)	(0.002)
SA		0.164^***^		
		(0.040)		
Risk				0.118^***^
				(0.016)
Controls	Yes	Yes	Yes	Yes
Year FE	Yes	Yes	Yes	Yes
Firm FE	Yes	Yes	Yes	Yes
cons	−3.728^***^	6.664^***^	0.965^***^	5.953^***^
	(0.159)	(0.208)	(0.114)	(0.206)
N	14019	13932	11800	11760
R^2^	0.100	0.684	0.062	0.664

#### 4.4.2. Examination of operational risk mediation effect.

Following the same principle as the examination of the financial risk mediation effect, the variable M representing operational risk (Risk) is selected as the mediating variable. Regression in Models (10) and (11) is performed to verify Hypothesis H4b. The results in Columns (3) and (4) of Table 6 demonstrate that in Column (3), the coefficient of the square term of corporate ESG performance is significantly positive at the 1% level, while the coefficient of ESG performance is significantly negative at the 1% level, indicating a U-shaped relationship between corporate operational risk and ESG performance. In Column (4), besides the quadratic term of ESG performance, the mediating variable Risk is included. The regression results reveal a significant positive correlation between the mediating variable Risk and audit pricing LnFEE. Even after incorporating the mediating variable, the regression coefficients of ESG^2^ and ESG remain significant, indicating that operational risk partially mediates the U-shaped relationship between corporate ESG performance and audit pricing. Hypothesis H4b is confirmed. In summary, our study verifies the non-linear relationship between ESG and audit pricing, while also considering the variations of this relationship under different conditions.

### 4.5. Robustness tests

#### 4.5.1. Shortening sample period.

In 2018, CSRC introduced a revised version of the Guidelines for Corporate Governance of Listed Companies, establishing an ESG disclosure framework that mandates listed companies to disclose information on environmental, social responsibility, and corporate governance in line with relevant laws and regulations. Hence, the study re-regressed Model (7) using samples post-2018, with results remaining statistically significant. Refer to Column (1) in Table 7 for specifics.

**Table 7:** Robustness tests.

	(1)	(2)	(3)	(4)
	LnFEE	LnFEE	LnFEE	LnFEE
ESG	−0.062^***^	−0.080^***^	−0.090^***^	0.786^***^
	(0.013)	(0.014)	(0.019)	(0.281)
ESG^2^	0.007^***^	0.007^***^	0.009^***^	−0.093^***^
	(0.002)	(0.002)	(0.003)	(0.033)
Controls	Yes	Yes	Yes	Yes
Year FE	Yes	Yes	Yes	Yes
Fim FE	Yes	Yes	Yes	Yes
cons	7.018^***^	5.779^***^	5.877^***^	4.239^***^
	(0.210)	(0.212)	(0.222)	(0.474)
N	6011	14272	5230	13624
R^2^	0.647	0.679	0.593	0.601

#### 4.5.2. Mitigating omitted variables.

To further alleviate potential biases from omitted variables, the study augmented related financial control variables: Book-to-Market ratio (BM), Inventory ratio (INV), Accounts Receivable ratio (REC), and additional corporate governance-related controls: Company Age (Age), Top Shareholder Ownership Percentage (Top1), and Board Size. Upon re-running Model (7), the results remained robust, as depicted in Column (2) of Table 7.

#### 4.5.3. Propensity score matching (PSM) treatment.

This paper employed the PSM method to address potential sample selection biases causing endogeneity. Specifically, setting ESG performance above the median as the experimental group and vice versa as the control, employing a nearest neighbor 1:1 matching, resulted in balanced mean deviations below 10%. No significant differences were observed between the experimental and control groups, indicating favorable matching effects. The regression post-matching reflected no alterations in the relationship between corporate ESG performance and audit pricing, as shown in Column (3) of Table 7.

#### 4.5.4. Endogeneity test.

Considering the potential influence of other regional corporate ESG performances on a company’s ESG performance, instrumental variables(IV) were employed. These variables were derived by excluding the current company’s ESG performance from the average ESG of other companies in the same year, industry, and province. The instrumental variable exhibited a significant positive correlation with the explanatory variable at the 1% level. The subsequent regression confirmed the unchanged U-shaped relationship between ESG performance and audit pricing. Detailed results are presented in Column (4) of Table 7, maintaining the study’s conclusions.

The robustness tests show that the findings are consistent across different models and methods, supporting the main regression results’ credibility. Based on this, the following text will summarize and discuss the study’s findings and limitations.

## 5. Discussion and conclusions

### 5.1. Results

In recent years, research on the economic implications of ESG has become more flourished. However, the exploration of how ESG performance affects audit outcomes remains in its early stages. Building upon prior research, this paper utilized Huazheng ESG ratings as the independent variable to explore its nonlinear influence on corporate audit pricing. The key findings are presented below:

(1) The impact of corporate ESG performance on audit pricing follows a U-shaped pattern. Initially, higher ESG scores often indicate that a company performs well in environmental, social, and governance aspects, thereby enhancing its brand image and social reputation [7]. Auditors may perceive companies with good reputations as lower-risk, and therefore be willing to offer lower audit fees. However, this effect is non-linear. As ESG performance improves, each additional increase has a diminishing impact on audit fees, although the effect remains negative. Once a critical point is reached, the relationship between ESG and audit pricing reverses, and the overall relationship becomes U-shaped. This conclusion remains valid after undergoing various robustness tests.
(2) The quality of internal controls moderates the relationship between ESG performance and audit pricing. Specifically, higher-quality internal controls flatten the U-shaped curve between ESG performance and audit pricing. Additionally, superior internal controls shift the turning point of the U-shaped curve to the right.
(3) Information asymmetry moderates the relationship between corporate ESG performance and audit pricing. Lower information asymmetry smoothens the U-shaped curve between ESG performance and audit pricing, while higher information asymmetry shifts the turning point of the U-shaped curve to the right.
(4) In the stage where ESG performance can reduce audit pricing, corporate financial risk, and operational risk play a mediating role. ESG performance reduces perceived risks (e.g., lower litigation likelihood from environmental incidents), which in turn decreases auditors’ effort allocation and fee premiums.

### 5.2. Policy implications

Drawing from these insights, the paper proposes several recommendations: Firstly, companies should prioritize ESG performance alongside economic gains, emphasizing sustainable development to enhance their ESG ratings. Emphasis should be placed on bolstering internal controls and reducing information asymmetry to maximize the mitigating effect of ESG on audit pricing. For instance, companies can establish ESG-specific committees within the board and set up ESG risk and compliance management systems. They can also incorporate ESG goals into performance evaluations, such as by linking executive pay to carbon reduction targets. Secondly, Chinese enterprises’ overall ESG disclosure is in an early stage, with an average score below the passing level, marked by insufficient completeness and a need for greater standardization. To address this, regulatory bodies should look to international sustainability disclosure standards and frameworks to guide businesses from voluntary to standardized disclosure. Establishing a framework for comparable ESG disclosure is essential. For instance, the standards of the Global Reporting Initiative (GRI) offer a comprehensive set of metrics and disclosure contents, which can be adjusted according to China’s national conditions. Thirdly, auditors should consider a company’s ESG during audit services. Integrating ESG information can enhance the efficiency and quality of financial report audits, aiding in audit planning and risk mitigation.

### 5.3. Limitations and future research

This paper is subject to certain limitations that could be addressed in future research for improvement. Primarily, the research sample focuses solely on Chinese listed companies, potentially restricting the generalizability of findings across diverse regions with differing market development, cultural contexts, and legal systems. ESG practices are shaped by national policies, industry norms, and cultural logic. Therefore, future research can explore differences in the ESG-audit pricing relationship under different legal systems, such as civil law (e.g., Germany) and common law (e.g., the US). This could involve examining how disclosure environments affect auditors’ perception of risk.

In addition, future research should further compare the impact of different disclosure environments (voluntary vs. mandatory) on audit pricing. It should analyze whether mandatory disclosure can effectively improve the quality and transparency of information, thereby reducing audit fees. Furthermore, As Huang et al. [57]have indicated, the voluntary disclosure of internal control reports may carry a certain degree of opportunism. The voluntary disclosure of ESG information can also lead to selective disclosure issues. This demands that auditors make judgments about additional information, such as management motives and governance risks. In this context, the study should explore whether ESG performance has a more complex effect on audit fees.

Finally, the study did not individually explore whether different E, S, and G pillars have varying impacts on audit pricing. Prior research indicates distinct economic consequences among these pillars, where the G pillar significantly affects profitability, while the S pillar holds the most substantial influence on corporate credit ratings [58]. Additionally, in risk mitigation, the E and G pillars play predominant roles [59–60]. Thus, the three distinct ESG pillars might exert differing effects on audit pricing and merit further investigation.
